# Extrinsic Regulation of Satellite Cell Function and Muscle Regeneration Capacity during Aging

**DOI:** 10.4172/2157-7633.S11-001

**Published:** 2012-09-26

**Authors:** JV Chakkalakal, AS Brack

**Affiliations:** 1 Center of Regenerative Medicine, Massachusetts General Hospital, Harvard Medical School, Boston, Massachusetts 02114, USA; 2 Harvard Stem Cell Institute, 135 Massachusetts Avenue, Boston, Massachusetts 02138, USA

**Keywords:** Muscle regeneration, Satellite cells, Skeletal muscle stem cells

## Abstract

Optimal regeneration of skeletal muscle in response to injury requires the contribution of tissue resident stem cells termed satellite cells. Normally residing at the interface between the muscle fiber and overlying basal lamina it is generally understood with age the satellite cell pool exhibits decline both in number and function. Over the past decade mechanisms that contribute to these declines have begun to emerge. Implicit in aged-related satellite cell dysfunction and decline is the involvement of signals from the environment. Many of the signals that become deregulated with age have conserved functions during distinct stages of muscle fiber formation both in early development and regeneration. In particular, modulations in Wnt, TGFβ, Notch and FGF emanating from aged skeletal muscle fibers or the systemic milieu have emerged as age-related alterations that significantly impact both the maintenance of the satellite cell pool and skeletal muscle regenerative efficacy. In this review we will summarize how the aforementioned pathways contribute to skeletal muscle development and regeneration.

We will then discuss deregulation of these cascades with age and how they contribute to satellite cell depletion and dysfunction. The review will also summarize some of the challenges we face in trying to draw parallels between murine and human satellite cell aging. Finally, we will highlight the few examples whereby FDA approved drugs may be exploited to modulate specific signaling cascades in effort to preserve skeletal muscle regenerative function with age.

## Introduction

During aging there is general decline in tissue maintenance and reparative function. Tissue resident stem cells are essential for the maintenance and regenerative capacity of adult tissues. As such, from mice to men and in other model organisms aging is associated with a loss in stem cell number and function [1–8]. However, our understanding of the mechanisms that lead to stem cell decline and how this contributes to tissue dysfunction with age is incomplete. Skeletal muscle is a paradigmatic system to study tissue aging. Composed of individual multinucleated muscle fibers containing a complement of quiescent resident stem cells termed satellite cells, skeletal muscle experiences major declines in function and regenerative capability with age [6,9–11]. The efficacy of skeletal muscle as a model system to study aging in large part stems from the accessibility and the capability to track both phenotypic and functional alterations of both muscle fibers and satellite cells. Many factors influence muscle fiber maintenance and regeneration, principal among them is the number and functional capacity of the satellite cell pool and the external signals that harness their potential [2,7,12–16]. In this review we will discuss our current knowledge of satellite cell function and skeletal muscle regeneration during aging. Specifically, we will focus on some signaling cascades that become altered with age and how they affect muscle regeneration.

Finally, we will highlight how basic research conducted on murine and human satellite cells has provided a platform to treat aging pathology.

## Maintenance of Skeletal Muscle Stem Cell Number and Function

In adult resting skeletal muscle, a complement of dormant satellite cells exists that reside adjacent to the muscle fiber and overlying basal lamina (i.e. the satellite cell niche). Although various morphological and molecular criteria have been employed, expression of the paired-box transcription factor 7 (Pax7) provides the most reliable marker of satellite cells in uninjured adult muscle fibers [17,18]. The functional importance of satellite cells to muscle maturation and regeneration is now beyond doubt [19]. Pax7-expressing satellite cells demonstrate stem cell potential based on extensive self-renewal and the ability to contribute differentiated progeny upon transplant [20,21]. Ablation studies using Cre-mediated diptheria toxin induction have shown a requirement for adult Pax7-expressing satellite cells for efficient muscle tissue repair and maintenance of regenerative potential [22–25]. Furthermore, these studies suggest that reductions in the numbers of satellite cells can delay or severely compromise skeletal muscle regenerative outcomes.

Aged skeletal muscle is associated with a notable decline in the number of Pax7+ satellite cells [7,26,27]. Despite these observations the mechanisms responsible for this decline are only just being uncovered. Assessment of aged satellite cell function through adherent cultures of muscle fibers demonstrates their propensity to progress through the myogenic program, however reduced numbers of cycling and differentiated progeny and the ability to return to quiescence, an indicator of renewal potential, is observed [27,30,31]. In a variety of tissues maintenance of the quiescent state or reversion back into quiescence after proliferation, is a mechanism utilized for the preservation of tissue-resident stem cell number and self renewal potential [29]. In both adult and aged resting muscle preservation of the quiescent state protects against satellite cell loss [27,28]. As we will discuss below, age-dependent change in the satellite cell niche are responsible for satellite cell loss.

Stem cell transplantation is considered the gold standard assay for testing cell autonomous stem cell potential. Adult and aged satellite cells have been compared under the setting of transplantation and yield contradictory results [27,30]. Engraftment of single aged muscle fibers with their associated satellite cells (n=~2) or flow cytometry-purified aged satellite cells (n=200) into irradiated, dystrophin-deficient (*mdx*) adult hosts, demonstrated extensive self-renewal and myofiber contribution [30]. In contrast, transplantation of larger numbers of aged satellite cells (n=2000) displayed an impaired function compared to adult satellite cells upon engraftment into non-irradiated wild type adult hosts [27].

The discrepancy in observations between these two studies may be a consequence of differences in methodology, primarily the influence of the host environment and donor cell number. We pose that in a less supportive host environment, for example after irradiation and on an *mdx* background, donor cells will have less competition from endogenous cells and thus unlock their potential. At the same time, decreasing donor cell number will increase the demand on each cell necessary to restore muscle homeostasis, thus increasing their ‘potential’ on a per cell basis. Hence, although aged satellite cells, as a population, may possess some cells with extensive regenerative capability there does appear to be a cell autonomous decline in function when aged cells are in competition with adult cells.

The manifestation of this cell autonomous decline may stem from the constant exposure of satellite cells to an aged niche, leading to inability to maintain a favorable quiescent state. Furthermore, the ability of the aged satellite cell population to seemingly revert to a younger state in a permissive environment may reflect the presence of subpopulations of satellite cells that are more adept to resist aged-related loss in quiescence [27].

## Efficacy of Regeneration

A hallmark of aged skeletal muscle is a delay in the regenerative process [6]. In response to degenerative stimuli muscle fibers express and release factors that induce satellite cells into cycle [32–34]. Blood-borne inflammatory cells rush to sites of injury to clear necrotic debris and function as a source of factors that influence satellite cell-derived muscle progenitor fate decisions [34]. As regeneration proceeds, the environment undergoes a transition from one that promotes muscle progenitor expansion to one that drives myogenic differentiation and the renewal of the satellite cell pool [34–36]. With age failures at various phases of skeletal muscle regeneration occur.

These failures lead to decreased muscle fiber formation, a void replaced by fibrotic or adipocyte tissue a hallmark of aged skeletal muscle [12,16,37,38].

Most of our knowledge to date suggests the systemic environment, the expression of factors from injured skeletal muscle and the responsiveness of aged satellite cells and downstream progenitors as factors that adversely affect aged skeletal muscle regeneration [26]. Heterochronic parabiosis, an experimental strategy to study the contribution of circulatory factors to tissue resident cell function, demonstrates that stimulatory components of adult serum and inhibitory components of aged serum modulate satellite cell function and skeletal muscle regenerative capacity [12,15].

Injured aged muscle fibers have been shown to express and secrete factors that inhibit satellite cell activation and expansion [13]. In addition, impaired sensitivity of aged satellite cells to mitogenic stimulation has also been observed [39]. While the direct affects of serum and injured muscle fibers on satellite cell activation, expansion and differentiation can be dissected in vitro, in vivo, additional cellular contributions notably from fibroblasts and other cells of non-satellite cell origin do influence regenerative outcomes [16,23,34,37,40].

## Extrinsic Regulation of Satellite Cells during Muscle Regeneration

Next we describe some signaling pathways known to participate in myogenesis and how they become deregulated with age. Furthermore, we will describe how these pathways influence aged skeletal muscle regenerative outcomes through direct influences on satellite cells or by modulating the activity of other cells present in regenerating skeletal muscle.

### Wnts

The Wnt signaling pathway has been implicated in various aspects of embryonic development, stem cell maintenance and tissue homeostasis [41–43]. In the canonical pathway, Wnt proteins bind to cell surface Frizzled receptors, which triggers the stabilization of the common downstream Wnt effector β-catenin [42]. Normally, β-catenin levels and nuclear accumulation are kept low due to continuous proteosome-mediated degradation driven by a destruction complex composed of glycogen synthase kinase-3β (GSK-3), Adenomatous Polyposis Coli (APC) and Axin [42]. Active Wnt signaling leads to disassembly of the destruction complex and resultant stabilization and nuclear accumulation of β-catenin. Subsequently, nuclear β-catenin enables target Wnt gene expression by promoting the activity of lymphoid enhancer-binding factor 1/T cell-specific transcription factor (LEF/TCF) proteins [42].

Wnt signaling has vital functions in early stages myogenic specification and muscle fiber formation. During embryogenesis the dermomyotome, which forms from the dorsal most aspect of somites, gives rise to muscle progenitors required for the formation of all trunk and limb skeletal muscles. Expression of Wnts 1, 3a from the dorsal neural tube and 7 from the ectoderm function as potent factors that promote cell precursors derived from the dorsal halves of somites to a myogenic fate [44–46]. Consistent with a role in myogenic specification, over-expression of Wnts at ventral portions of the neural tube near the notochord is sufficient to expand the boundaries of myogenic precursor production to more ventral aspects of somites [46].

The role of Wnts as myogenic commitment factors is also observed during adult regeneration. Satellite cell muscle fiber explant cultures demonstrate increases in Wnt3a expression and Wnt signaling during stages of myogenic terminal commitment [35]. In vivo, supplementation of Wnt3a during regeneration accelerates whereas inhibition of Wnt signaling inhibits myogenic lineage progression of muscle progenitors [35]. The effects of Wnt knockdown could only be observed during stages of myogenic commitment. Consistent with an important role for Wnt signaling in myogenic commitment, knockdown and deletion of a downstream mediator of Wnt signaling, BCL9, is sufficient to prevent myogenic commitment of muscle progenitors during regeneration [47]. Hence, Wnt signaling is necessary to ensure efficient myogenic lineage progression of progenitors and optimal skeletal muscle regeneration.

With age rigid regulation of Wnt signaling is lost. Indeed, parabiosis experiments have clearly demonstrated elevated Wnt activity in serum from aged mice [12]. The factor(s) responsible for elevated Wnt signaling was not identified in this study, however depletion of serum factors that bind to family members of Frizzled receptors reduced Wnt signaling of aged serum to that of adult levels, implicating if not a Wnt family member, a Frizzled binding ligand. Recently, complement C1q was identified as a factor found at elevated concentrations in aged serum that binds to Frizzled receptors and stimulates canonical Wnt signaling in both fibroblasts and satellite cells [16]. Elevated aged serum-derived Wnt activity promoted the proliferation of fibroblasts and had pleiotropic affects on satellite cells. Indeed, in addition to inhibiting the proliferative response of satellite cells, lineage trace analysis revealed that aged serum could direct the fate of satellite cells towards a fibrogenic lineage [12,16]. Many of these phenotypes were abrogated when aged serum had been immunodepleted of Wnt activity or obtained from Clq1 deficient mice [12,16]. Furthermore, deletion of Clq1 and the injection of the Wnt inhibitors DKK1 and sFRP3 led to reductions in fibrotic accumulation and non-myogenic cells observed in aged regenerating muscle [12,16].

Hence, elevated Wnt signaling from aged serum promotes increased fibrotic accumulation through multiple cellular mechanisms including defective satellite cell function.

### TGFβ

The TGFβ signaling pathway is involved in a variety of cellular processes in mature and developing organisms [48]. Ligands of the TGFβ superfamily bind to type II receptors that subsequently homodimerize and phosphorylate type I receptors. As a result type I receptors also homodimerize. A tetramer complex composed of type I and II homodimers forms which through intracellular modifications induces the phosphorylation of receptor SMADs 2/3 (R-Smads). Phosphorylated R-Smads 2/3 then associate with SMAD4, to form a trimetric complex. These trimetric SMAD complexes then accumulate in the nucleus where together with chromatin modifiers and other transcriptional co-factors promote the expression of target genes [49].

Members of the TGFβ family of growth factors can affect a variety of cell types that influence the development and regeneration of skeletal muscle. The expression of TGFs has been observed in peripheral portions of developing lower limb muscles, at low levels in mature muscle fibers and at high levels during early stages of muscle fiber regeneration and other cell types in skeletal muscle [50–53]. TGFβ signaling is generally associated with inhibition of myogenic differentiation. During embryonic development muscle formation involves the sequential activation and proliferation of at least two distinct populations of myoblasts embryonic and neonatal that give rise to primary and secondary muscle fibers respectively. Using limb organ and myoblast cultures the Cossu group demonstrated that neonatal myoblasts, unlike embryonic, were sensitive to TGFβ signaling [54,55]. Specifically, supplementation of neonatal, as opposed to embryonic, cultures with TGFβ prevented their ability to form terminally differentiated myosin heavy chain positive myotubes in culture [54,55]. Conversely, neutralization of TGFβ in organ limb cultures was sufficient to induce, although reduced in number, the earlier appearance of larger neonatal-like myotubes [55]. Similar to neonatal muscle progenitors, treatment of human and rodent satellite cell preparations with TGFβ is also associated with a reversible inhibition of myogenic differentiation and reduced proliferation [56–58]. Consistent with a requirement for TGFβ signaling in satellite cell maintenance, SMAD3 mutants display severely impaired regeneration concomitant with a reduction in Pax7 expressing satellite cells both prior to and after injury [59,60].

An elevated level of circulating TGFβ occurs with age [61]. It has been proposed that aged-related elevations of TGFβ signaling impair satellite cell entry into cycle and the transient expansion of downstream muscle progenitors. Consistent with elevated TGFβ signaling, phospho-SMAD3 levels are increased in aged compared to young satellite cells [13,62]. Indeed, administration of TGFβ to cultured satellite cells promotes the association of phospho-SMAD3 with promoters for the cell cycle inhibitors p15, p16, p21 and p27 thereby directly inducing their expression [13]. Furthermore, direct intramuscular injection to sites of muscle injury with neutralization antibodies to TGFβ and lenti viral particles containing shRNAs to SMAD3 rejuvenated muscle fiber formation in aged regenerating skeletal muscle [13]. However, the direct affects of TGFβ signaling on satellite cell function in an aged environment are unclear. When supplemented with both low and high dosages of TGFβ cultures of aged satellite cells can readily form myotubes. In contrast, TGFβ supplementation severely compromises the ability of young satellite cells to form myotubes [62]. Hence, although TGFβ signaling is elevated in aged satellite cells the ability of this pathway to negatively impact aged muscle fiber repair may reflect cell intrinsic differences between adult and aged satellite cells and their progeny, in addition to influences of TGFβ on additional cell types in vivo.

Increased fibrosis and adipogenesis are hallmarks of various dystrophies and aged muscle repair, processes known to be enhanced by TGFβ signaling [12,16,37,63]. Recently, in δ-sarcoglycan deficient mice (Sgcd−/−) a model of limb girdle muscular dystrophy, TGFβ signaling was shown to drive the expression of periostin, a matrix protein produced specifically by interstitial fibroblasts that perpetuates fibrosis [63].

Interestingly, Sgcd−/− mice bred to a perisostin null background had reduced fibrosis together with much improved muscle physiology and regenerative capability. Notably, the beneficial affects of perisostin deletion on muscle physiology and repair were mitigated by inhibition of TGFβ signaling [63]. Hence, although elevated TGFβ in a diseased context promotes fibrosis this pathway is required to ensure appropriate muscle fiber function and repair. It will be of interest to determine how periostin deletion may affect aged muscle fiber physiology and regeneration.

### Notch

Notch signaling is primarily elicited through interactions of cognate receptors (Notch1–4) with membrane bound ligands. Ligand binding to Notch receptors leads to proteolytic cleavage events that release an intracellular domain. Upon release the Notch intracellular domain (NICD) enters the cell nucleus where interactions with the transcriptional regulator RBP-Jk lead to the recruitment of additional co-factors enabling Notch target gene expression [64,65]. The Notch pathway has essential roles in the maintenance of muscle progenitors during muscle development. Conditional deletion of RBP-Jk in migrating or non-migrating muscle progenitors with Lxb1 or Pax3 Cre drivers respectively leads to depletion of myogenic progenitors due to precocious terminal differentiation [66]. Mice heterozygote or hypomorphophic for the Notch ligand Delta1 also promote depletion of myogenic progenitors due to premature differentiation [67]. Consistent with premature loss of muscle progenitors genetic inhibition of Notch signaling results in hypertrophic skeletal muscles and depletion of satellite cells in the late embryo [66,67].

Notch signaling also has vital roles in the maintenance of muscle progenitors during adult regeneration. Injection of NICD into adult regenerating muscle maintains myogenic progenitors by preventing terminal commitment [32]. In support of this observation, specific over-expression of NICD in satellite cells and downstream progenitors inhibits myogenic differentiation during adult regeneration [32,68]. Conversely, injection of the Notch inhibitor Numb promotes premature differentiation and a corresponding depletion of myogenic progenitors [32].

In response to injury aged, in comparison to adult, skeletal muscle demonstrates a reduced ability to promote Notch signaling [14]. Parabiosis experiments revealed an inability of aged serum to induce increased expression of the Notch ligand Delta1 from skeletal muscle fibers and satellite cells [15]. The inability of aged serum to induce notch signaling was shown to impede expansion of both young and aged muscle progenitors in a regenerating environment. Impaired transient amplification of progenitors in the presence of aged serum can be rescued in part through activation of notch signaling by injection of extracellular fragments of Notch1 [14,15]. Therefore, transient stimulation of Notch signaling provides a means to increase progenitor numbers to enhance aged skeletal muscle regeneration.

In addition to its role in progenitor expansion, active Notch signaling has recently been proposed as a means to ensure satellite cell quiescence [69,70]. Specifically, satellite cell-specific deletion of RBPJκ leads to loss of satellite cell quiescence in resting adult muscle. Whether deregulation of Notch signaling participates in age related decline is not known. However it is worth considering that due to diminished levels of the Notch ligand, Delta1 in aged muscle fibers [14], the signaling pathways that control satellite cell number may differ in adult and aged muscle.

### Fibroblast Growth Factor (FGF)

FGFs are well-characterized mitogens that induce satellite cells into cycle [57,71]. There are four FGF receptor subtypes, which can be activated by more than twenty-three different FGF ligands [72,73]. Roles in myogenesis and muscle regeneration have been ascribed to receptors FGFR1 and 4, whereas the expression FGFRs 2 and 3 have been documented to be low or undetectable in myogenic cells [71,74,75].

Inhibition of FGF signaling using FC-receptor binding fragments or dominant negative FGFR variants has been shown to negatively impact muscle mass. Although not entirely clear, the mechanisms for the reduced muscle mass seen upon inhibition of FGF signaling have been attributed to depletion of muscle progenitors as a consequence of premature terminal differentiation [76–78]. Further substantiating a role for FGF signaling in myogenic commitment, over expression of sprouty proteins, negative feedback regulators of FGF signaling, biases proliferating embryonic muscle progenitors to a self renewing fate at the expense of differentiation [79].

Genetic studies have shown roles for FGF signaling during adult muscle regeneration. Mice null for FGFR4, FGF6 and both FGF6/FGF2 display impairments in muscle regeneration due to defective satellite cell activation and migration [75,80,81]. However, prolonged stimulation of FGF signaling is detrimental to satellite cell renewal.

Satellite cell specific deletion of sprouty1 leads to sustained ERK activity that drives subsets of muscle progenitors to apoptosis [36]. Hence, temporal strict control of FGF signaling is required to ensure efficient regeneration of muscle and renewal of satellite cells. Consistent with impaired activation aged satellite cells have reduced sensitivity to FGF signaling [39]. The molecular basis for reduced sensitivity of aged satellite cells is unclear. Recently aged muscle fibers have been shown to express high levels of FGF2 [27]. Therefore, one possibility involves receptor mediated down regulation of FGFRs, a common mechanism employed by cells to desensitize them to the presence of excess ligand.

Elevations in muscle fiber derived FGF2 has been demonstrated to disrupt satellite cell quiescence leading to diminution of the satellite cell pool [27]. This result initially appears to be at odds with previous observations examining crushed muscle extracts. Examination of crushed aged skeletal muscle extract isolated in a saline solution revealed little or no mitogenic activity [82]. In a modification of this procedure, enzymatically liberated and repeatedly washed single muscle fibers were incubated in a high salt extraction buffer to remove any receptor and extracellular matrix-bound ligands from skeletal muscle fibers [27]. The modified procedure revealed the presence of satellite mitogenic activity in aged muscle fiber extracts. Notably, this activity could be blocked by; neutralization of FGF2, pharmacological inhibition of FGFRs and genetic deletion of FGFR1. Hence, increased expression and activity of FGF2 from aged muscle fibers indicates that the aged niche drives the loss of satellite cell quiescence. Interrogation of the aged satellite cell pool revealed subpopulations that were more adept in retaining quiescence and function [27]. The relatively dormant aged satellite cells express higher levels of the quiescent marker Sprouty1, a negative feedback regulator of FGF signaling previously implicated in satellite cell renewal [36]. Satellite cell specific deletion of Sprouty1, in the aged niche led to further loss in quiescence and reductions in pool size, which severely affected skeletal muscle regenerative capability.

Therefore, FGF feedback regulation provides an important means to sustain satellite cell quiescence and pool size. Importantly, deletion of Sprouty1 did not disrupt quiescence of adult satellite cells in resting muscle, consistent with the accumulation of FGF2 specifically in the aged satellite cell niche. Therefore regulation of satellite cell number is age-dependent, governed by the level of niche-derived ligands in the aged muscle.

### Cross-talk

In response to injury the coordinated activation of satellite cells, expansion of downstream progenitors and provision of differentiated progeny is required for optimal regeneration. Such coordination requires efficient transitions between signals that promote different aspects of muscle regeneration. Initially, transient expansion of muscle progenitors requires Notch signaling [32,35]. During this initial phase of transient expansion Notch signaling actively inhibits Wnt action. Inhibition of Notch signaling by treatment of satellite cells stripped from muscle fibers with γ-secretase inhibitor results in premature Wnt activity [35]. Notch signaling also promotes the phosphorylation of GSK3-β on residues that drive the activity of this kinase thus promoting the degradation of β-catenin [35]. Therefore, GSK3-β functions as a key nodal point that coordinates transitions between the proliferative actions of Notch and differentiation promoting canonical Wnt signaling.

During the initial stages of regeneration phospho-SMAD3 functions as a nodal point whereby Notch antagonizes whereas TGFβ promotes the expression of cell cycle inhibitors in muscle progenitors [13]. Forced activation of Notch signaling in the presence of TGFβ attenuates increases in the expression of cell cycle inhibitors. Under these circumstances active Notch directly associates with phospho-SMAD3 displacing it from the promoters of cell cycle inhibitors [13]. Thus, in an aged environment reduced Notch and elevated TGFβ activity collaborate to promote the expression of cell cycle inhibitors. Therefore, signaling cross-talk is an important mechanism that ensures efficiency in the regenerative response. Considering the multiple pathways involved at distinct stages of myogenesis it is likely that other modes of cross-talk exist. The identification of other examples of crosstalk will inevitably require the characterization of nodal points such as GSK3-β or SMAD3 whereby distinct signaling cascades may converge.

### Human satellite cell studies

Most of our understanding of satellite cells comes from studies on murine models, hindered by the difficulty in obtaining sufficient quantities of human material for satellite cell isolation and analysis. It is essential we complement studies on model organisms with studies conducted on human samples. Comparison between adult human and mouse muscle suggests a similar percentage of satellite cells, identified by electron microscopy, between each species [83,84] Human satellite cells can be identified by expression of Pax7 and CD56 (NCAM) [85]. Immunotypic marker analysis indicates that the number of satellite cells declines during human aging [86,87]. Moreover, CD56+myogenic cells isolated from young and adult patients engraft in irradiated immune compromised mouse muscle and are capable of self-maintenance and differentiation [88]. Thus providing a tractable assay to test functional capacity of human satellite cells in the context of aging or other muscle diseases.

Similar to aged mouse muscle, human skeletal muscles displays reduced expression of the Notch ligands Jagged1 and Delta like 1 [86,89]. Correspondingly, aged human satellite cells have diminished Notch activity [86]. In addition, aged satellite cells display reduced phospho-ERK consistent with reduced mitogen signaling. However the ability of the systemic environment to influence satellite cell phenotype, assayed by culturing human satellite cells with human sera has yielded contradictory results [86,90].

As our ability to obtain pure populations of sufficient numbers of human satellite cells improves, the critical regulatory components that are common and distinct between different species will become apparent.

### From bench to bedside

Over the past decade there have been numerous insights into aged skeletal muscle regeneration and satellite cell maintenance. However, translation of this knowledge into effective therapeutic regimens designed to attenuate aged-related declines in muscle function and mass remains a challenge. Recently, Losartan an FDA approved drug that functions as an angiotensin II receptor antagonist to treat high blood pressure has been shown to attenuate fibrotic build up in regenerated aged muscle fibers [37]. The decrease in fibrosis observed upon Losartan treatment correlates with decreased TGFβ signaling in whole skeletal muscle protein extracts. In addition Losartan has been shown to attenuate accelerated atrophy of aged skeletal muscle in response to immobilization. In this context, Losartan treatment of immobilized aged skeletal muscle correlated with elevated IGF signaling with no changes in TGFβ signaling. Hence, depending on the model used Losartan was shown to promote muscle fiber recovery through different pathways [37]. However, the cellular mechanisms whereby Losartan improves aged skeletal muscle physiology in these injury models remains unclear. Specific dissection of the cellular and molecular mechanisms whereby Losartan or similar pharmacological interventions can improve aged skeletal muscle repair will be required.

Pyrvinium, an FDA approved small molecule drug, was recently identified in a chemical screen as a factor that prevents Axin degradation [91]. Subsequent analysis revealed Pyrvinium targets Caesin Kinase 1α a molecule that functions with GSK-3β to promote β-catenin degradation. Specifically, Pyrvinium promotes Casein Kinase 1α activity thus inhibiting canonical Wnt signaling. Through the use of induced myocardial infarction, a model of myocardial repair, administration of Pyrvinium was shown to promote cellular proliferation at sites of injury and beneficial cardiac remodeling [91]. It will be of interest to determine if pyrvinium may accelerate and promote beneficial aged skeletal muscle repair in response to injury. Collectively, these studies suggest that interventions through re-purposing of FDA-approved drugs or identification of novel small molecules, may present viable strategies to harness the functional potential of human satellite cells.

## Conclusion

**Figure 1:**
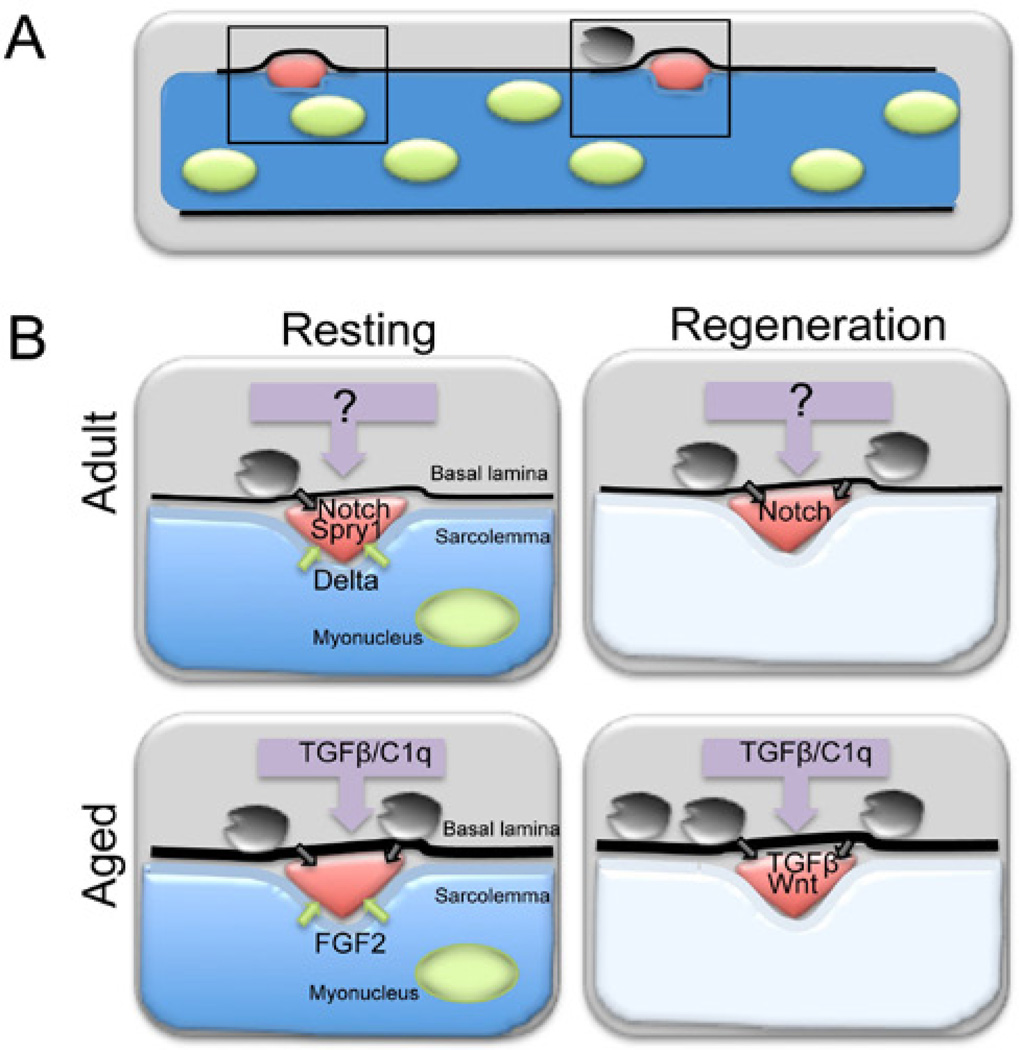
Age-dependent changes in extrinsic signaling cascades between the satellite cell and its microenvironment during homeostasis and early response to injury (A) Satellite cells (red) residing between the sarcolemma and the basal lamina of multinucleated (green) skeletal muscle fibers (blue). Other cells such as fibroblasts (black) are present outside the lamina. The systemic environment is represented as a grey box surrounding the muscle fiber. Areas highlighted with black boxes are shown in B. (B) Adult (top row) and aged (bottom row) muscle in resting (uninjured, left column) and regenerating (right column) conditions. In adult resting muscle (top left) the Notch ligand Delta1 is expressed in satellite cell microenvironment Notch1 receptor is expressed in quiescent satellite cells. Spry1 the downstream target and intracellular FGF inhibitor is robustly expressed in quiescent satellite cells. The ligands necessary for Spry1 expression in quiescent satellite cells remain unidentified. In response to injury (right column) the muscle fiber degenerates (white) adult (top right) satellite cells signal via Notch to promote progenitor proliferation. Factors from the adult systemic environment are stimulatory to satellite cells (purple arrow). The stimulatory factors remain unidentified. In resting aged muscle (bottom left) Delta1 declines and FGF2 increases in the aged satellite cell microenvironment, leading to diminished levels of Spry1 and Notch signaling. In resting muscle, aged satellite cells are more mitotically active compared to adult satellite cells. The aged systemic environment (purple arrows) contains increased levels of TGFβ and Complement 1q (C1q) that hinders satellite cell activation and increases fibrogenic conversion of satellite cells. In injured aged muscle (bottom right), due β to decreased Notch activation and increased Wnt and TGF signaling aged satellite cells activate poorly. A fraction of satellite cells acquire a fibrogenic fate. Fibroblasts or other cells outside the basal lamina in the microenvironment may also provide signals (black arrows) that influence satellite cell contribution to muscle repair.

It is beyond doubt that muscle regeneration capacity declines during aging, in large part due to loss in satellite cell number and function. As we unravel the inherent complexity within regenerating tissues that exist to maintain homeostasis we reveal the multiple levels of regulation from the cell autonomous to non-cell autonomous including the systemic environment and microenvironment that perturb cellular outcome during aging (Figure 1). In the near future we believe great strides will be made in our understanding of human satellite cells, this is essential to transition from “bench to bedside” to ameliorate the effects of aging on human regenerative potential.
